# Community resilience through partnership after the Great East Japan Earthquake: cooking classes by Iwate Co-op and a food company

**DOI:** 10.3389/fpubh.2024.1414480

**Published:** 2024-08-01

**Authors:** Naomi Ito, Kayoko Konno, Kumiko Nozaki, Kumiko Fukushi, Kasumi Kanno, Hiromi Kawamura, Yayoi Nakamura, Mikio Yamada, Ai Kuroda, Toshiya Kuchii, Yuri Kinoshita, Teru Nabetani, Yoshiharu Fukuda

**Affiliations:** ^1^Department of Radiation Health Management, Fukushima Medical University School of Medicine, Fukushima, Japan; ^2^Miyako Co-op, Iwate Co-op, Miyako, Japan; ^3^Kamaishi Co-op, Iwate Co-op, Kamaishi, Japan; ^4^Kesen Co-op, Iwate Co-op, Ofunato, Japan; ^5^Organization/Management Headquarters, Iwate Co-op, Takizawa, Japan; ^6^The Ajinomoto Foundation, Tokyo, Japan; ^7^Teikyo University Graduate School of Public Health, Tokyo, Japan; ^8^Red Apron Project Evaluation Team, Sendai, Japan; ^9^Tohoku Seikatsu Bunka Junior College, Sendai, Japan; ^10^Faculty of Human Health, Kurume University, Kurume, Japan

**Keywords:** Great East Japan Earthquake, cooking class activities, Ajinomoto Foundation, Iwate Co-op, community resilience, post-disaster

## Abstract

**Introduction:**

This case study aimed to demonstrate how cooking class activities held in collaboration with the Ajinomoto Foundation (TAF) and a consumer cooperative after the Great East Japan Earthquake contributed to the resilience of the affected community.

**Methods:**

With reference to the logic model, evaluation indicators for the case study were established. We focused on the Iwate Seikatsu Kyodo Kumiai (Iwate Co-op). We organized 120 continuation cases out of the eight-and-a-half-year activity records of the project owned by TAF (April 2012 to March 2020). The Iwate Co-op was one of the 120 continuing cases and had special features, including awareness that its members were responsible for the community.

**Results:**

The collaboration revealed three effects. First, owing to the encounter and collaboration with TAF, the cooking class was continuously conducted even after TAF withdrew from the disaster-affected areas. Second, the Iwate Co-op trained some of its members as food support staff to run the cooking class independently and was actively involved in obtaining the necessary budget for the operation, consequently leading to the independent activation of member activities. Third, they developed a cooking class project in inland areas other than disaster-stricken areas, assuming that they could incorporate the project into their existing activities, as food problems affected people beyond disaster victims.

**Conclusion:**

The collaborative food support project of the Iwate Co-op and TAF contributed to the resilience of the affected people and communities by strengthening bonds and solidarity among residents and organizations. The key to success was the fusion of a traditional sense of independence in the co-op with TAF’s mission and technical know-how. The partnership between the Iwate Co-op and TAF allowed the former to aim toward developing food support activities in the affected areas, accelerating the resilience of the community in the Iwate Prefecture.

## 1 Introduction

Community resilience refers to the process by which a community returns to its original or an improved state after being affected by a disaster or crisis (1–3). The community’s culture, traditions, bonds, and information-sharing inclinations accelerate this process; the bonds and solidarity within a community determine the speed and effectiveness of resilience. Communities with strong ties and solidarity can respond effectively and recover quickly, even in difficult situations (3).

The massive earthquake and tsunami that struck Eastern Japan in March 2011 left 22,318 people dead or missing (4). Following the accident at the Tokyo Electric Power Company’s Fukushima Daiichi Nuclear Power Plant (F1NPP), more than 380,000 people were forced to evacuate a week later. Owing to various circumstances, approximately 30,000 people are still unable to return home (as of December 2023) (4). Communities were destroyed, especially in the coastal towns of the three Tohoku prefectures (Iwate, Miyagi, and Fukushima), and the people in the region lost their local intimate networks. Thus, mental and physical care for the affected people is necessary. However, rebuilding the lives of older adults and addressing their isolation are challenging endeavors (5, 6).

Previous research on disaster resilience in aging populations by Kawachi et al. (7) emphasized the critical role of social connections (the “social capital” of a community) in enhancing disaster resilience by restoring the social fabric of people’s lives. Additionally, Tani et al. (8) reported that improving cooking skills may be key to boosting social relationships and social capital, which would prevent social isolation among older adults in Japan. In another systematic review, Hasan et al. (9) reported that cooking class interventions were associated with improved attitudes, self-efficacy and a healthier dietary intake in adults and children. However, there is currently a lack of research on the continuing of cooking classes post-disaster.

Even in Japan, a developed country, older adults people who have lost their own land, homes, property, and networks of people after a catastrophe face the risk of food insecurity (10). The Food and Agriculture Organization of the United Nations (FAO) describes food insecurity as consisting of four components: Availability (quantitative sufficiency), Access (physical and economic availability), Utilization (appropriate use taking into account nutritional aspects, etc.), and Stability (food is always available) (11). Older survivors often have problems with Access and Utilization, and Fujimoto et al. report that older people living in temporary housing are reluctant to cook meals because their kitchens are small, they lack cooking utensils, and they do not feel like cooking meals or find it troublesome to do so (12). In addition, in public disaster housing, where disaster victims who have been forced to relocate live, some individuals are unable to grow their own vegetables in the fields as they used to, or lack the availability of transportation to grocery stores, making access to food difficult. Kinoshita et al. reported that nine-and-a-half years after the disaster, the food intake diversity score (DVS) of older adults people in disaster public housing tends to be low, indicating the need for nutritional and dietary support (13). Food insecurity after a catastrophe is not only an immediate post-disaster issue, but also a mid-to long-term challenge.

**Figure 1:**
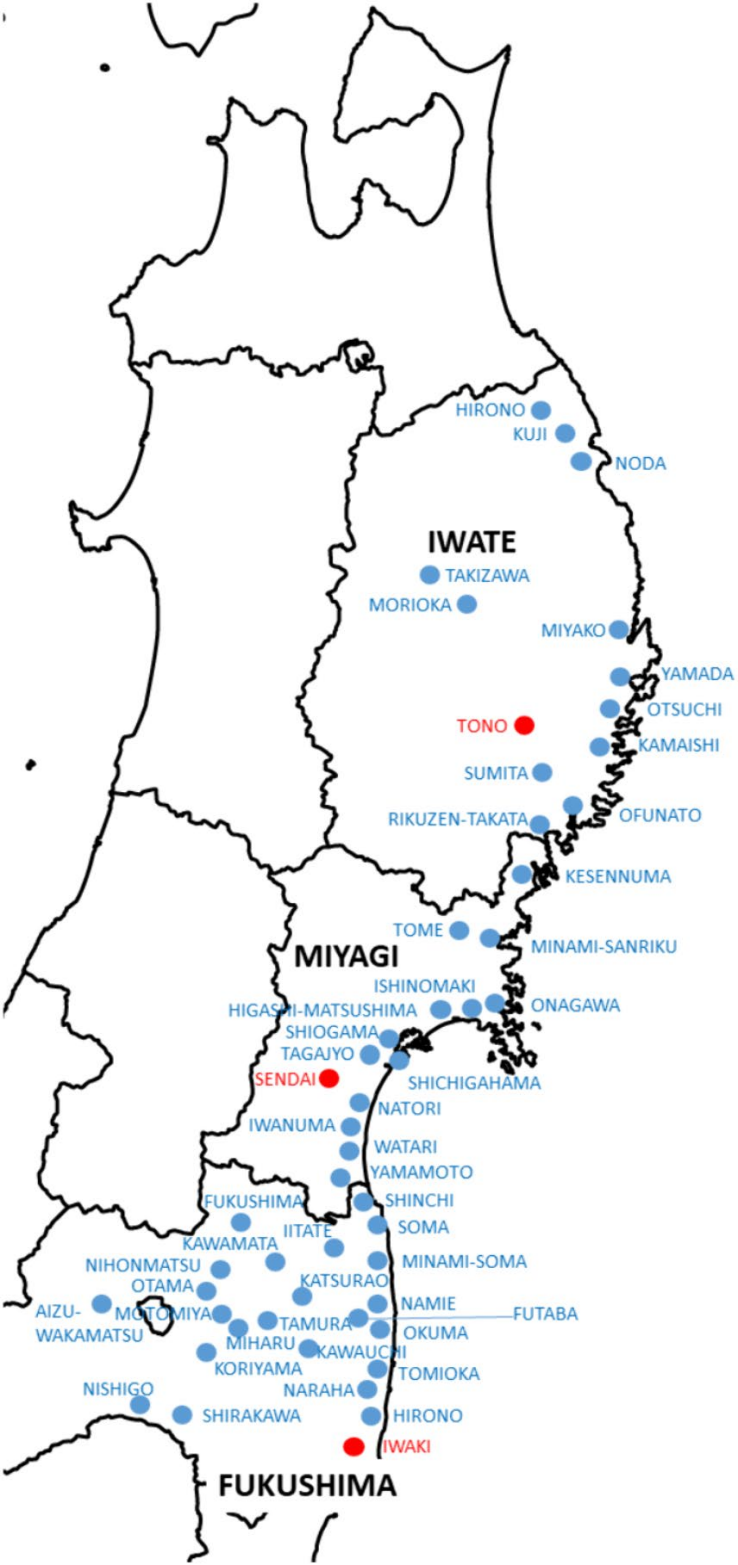
“Red Apron Project” for the Great East Japan Earthquake Reconstruction Support Map of municipalities where we held seminars between October 2011 and February 2020 (51 municipalities in Iwate, Miyagi, and Fukushima). TAF had 3 bases (red dots) and continued its cooking class activities in 51 locations (blue dots). This map was created by TAF.

**Figure 2:**
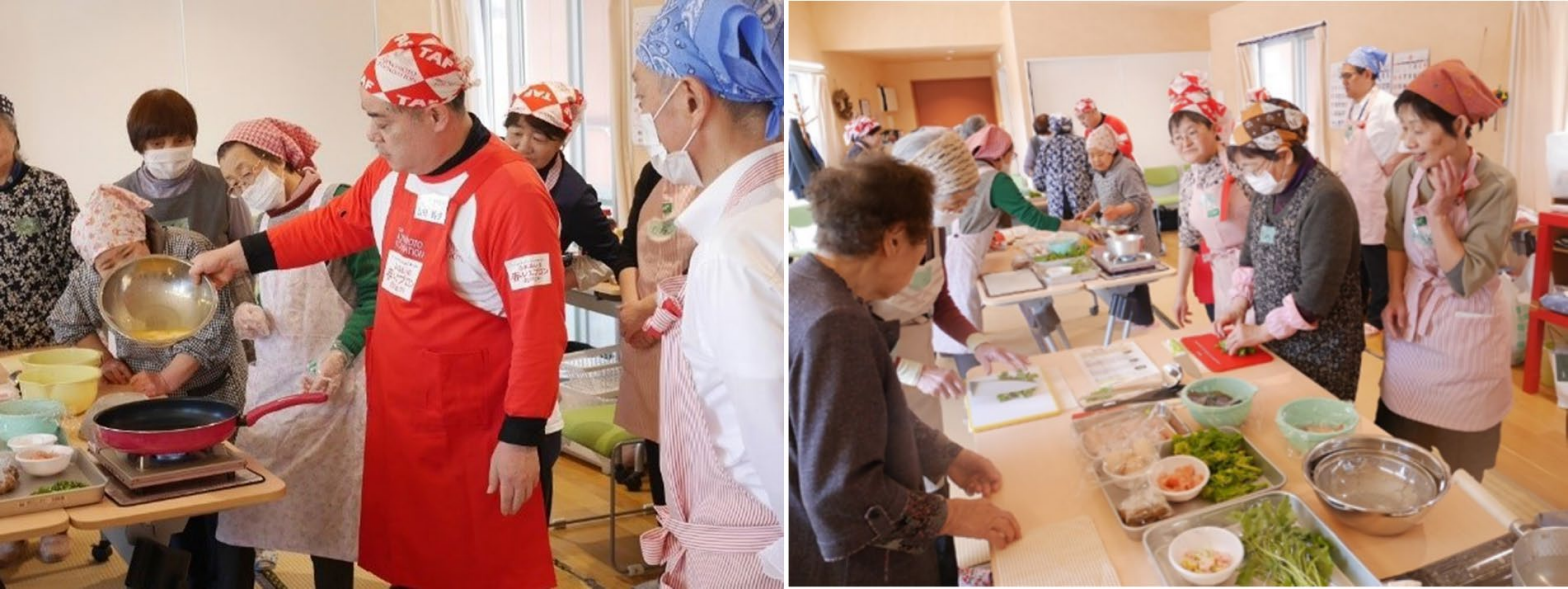
Cooking class: TAF staff give a demonstration (left), and all participants cook together (right). The activities of Iwate Co-op were photographed by a TAF staff member.

Under these circumstances, the Ajinomoto Group (14–16), a food company, launched a disaster recovery project in October 2011 with on-site staff to address the need to not only improve the dietary and nutritional status of disaster victims but also rebuild and revitalize the local communities destroyed by the disaster (17). The Ajinomoto Group and partner organizations across the three Tohoku prefectures, including the local government, social welfare councils, private organizations, neighborhood associations, and universities, conducted outreach cooking classes under the name “Fureai Red Apron Project” (Figure 1) (17). The concept was “Let us cook together and eat together” (Figure 2). Cooking classes were held 3,771 times in 51 cities, towns, and villages, with 54,434 participants (17). The Ajinomoto Group transferred this project to the Ajinomoto Foundation (TAF) in 2017 and provided direct local support until March 2020, after which it provided logistical support (18). We refer to the Ajinomoto Foundation as TAF hereafter.

The purpose of this study is to reveal how the cooking class activities, which were continuously undertaken by several organizations in collaboration after the Great East Japan Earthquake, contributed to the resilience of the community. This study addressed the lack of research on the impact of post-disaster cooking classes on community resilience. It will contribute to policy, research, and practice in the areas of public health and nutrition by highlighting the benefits of partnerships after disaster.

## 2 Methods

Of the eight-and-a-half-year activity records of the project led by TAF (April 2012 to March 2020), we organized 120 continuation cases. We focused on the Iwate Seikatsu Kyodo Kumiai (Iwate Co-op). This resident-led organization acquired the know-how of cooking classes for disaster victims at the earliest stage of the project and became self-supporting without assistance from TAF. This case study highlights how the organization has contributed to the resilience of the region.

With reference to the logic model, evaluation indicators for the case study were established. The following indicators were set as inputs: human resources, materials, and funds needed to operate the program; implementation and role assignment of the cooking class as a process; number of times the program was implemented, duration of implementation, and number of participants as outputs; stable food security and change in peer relationships as short-term outcomes; impact on food awareness and health behaviors; and improvement in food security (Utilization). The long-term outcomes were community revitalization and strengthening of community resilience. The activities were described around these outcomes, and the effects of the activities were analyzed.

## 3 Data collection

We directly interviewed the person in charge of the project at the Iwate Co-op (19) in July 2020 and asked for input from the board members in August 2022. Additionally, we conducted a case review and additional interviews via phone and email in August 2023. This study was approved by the ethics committees of Tohoku Seikatsu Bunka University and Junior College (approval no. R4-14). Written informed consent was obtained from the Iwate Co-op for the publication of this case study.

## 4 Context

### 4.1 Outline of Iwate Co-op

A consumer co-op (20) is a nationwide autonomous association of consumers united voluntarily to meet their everyday needs and aspirations in Japan, aiming at “a community where people live with a sense of security” and “a sustainable world where no one is left behind.” The Iwate Co-op is a local organization with the same purpose and is based in Takizawa City, Iwate Prefecture (19). It has 267,000 members, representing 50.7% of all the families (as of the end of 2020), and half of the families in the prefecture are members (Iwate Prefecture population: 1.21 million, 530,000 households) (21).

Immediately after the earthquake, the Iwate Co-op started providing mutual aid and assistance such as lending mourning clothes to those who had lost everything in the tsunami. They continued four significant activities to support reconstruction: “shopping support,” “support for making a living,” “activities to bring smiles and cheer,” and “activities not to let the disaster fade away.” Among them, “activities to bring smiles and energy” mainly encompassed not only salon activities including lunchtime meetings and tea parties but also the distribution of “three-line recipes” to motivate those living alone to live more wisely in temporary housing (22, 23).

### 4.2 Background of starting cooking classes for disaster victims

Iwate Co-op had been continuing the salon activities in the temporary housing facility and recognized the necessity of “food support” to meet the needs of the victims. Gradually, professional skills were required for cooperative activities. However, there were no food specialists, such as dietitians, available. Around that time (2016), they learned that TAF and the Council of Social Welfare held cooking classes almost daily in various locations along the coast. Notably, TAF was looking for a suitable partner so as to hand over the know-how of its activities to a local organization, as it would eventually withdraw from the disaster-affected area.

**Figure 3:**
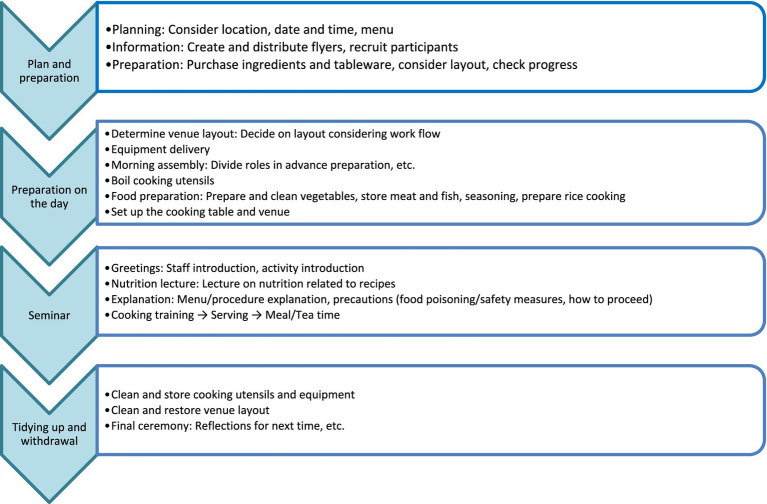
Cooking class seminar flow. This was provided by TAF.

Iwate Co-op was aware that its members were responsible for the community and believed that sooner or later, “local people ought to work for the local community” rather than having someone else from outside continue the project for them. It was especially beneficial for the Iwate Co-op to take over the know-how and activities of TAF’s cooking classes, which had already achieved successful results earlier, as they hoped to expand their “food support” activities in coastal areas. The cooking class was a suitable activity for them to continue (Figure 3), even after relocating from temporary to public housing. As it turned out, Iwate Co-op’s philosophy was congruent with TAF’s goal.

In September 2016, the Iwate Co-op and TAF started a “cooperative cooking salon” at three locations (Miyako, Kamaishi, and Kesen). Each location held the salon once a month at a meeting place for the reconstruction of public housing or a community center. Iwate Co-op assigned one “food support staff member” and two to five assistant staff members to each location to organize and manage the event. TAF was responsible for preparing ingredients, cooking utensils, and recipes, as well as providing lectures, and their recipes were simple, lean, and easy to follow. After 2017, the cooperative gradually shifted to classes run solely by its members and became a self-supporting activity by 2020. After becoming self-supported, they set a participation fee for their activities. One challenge confronted by the Iwate Co-op was the development of new project staff members available to take over the role of TAF. They set up a “food lover meet” and asked people who loved to eat, cook, and serve meals to join the staff. TAF trained and supervised new staff members to teach cooking. Eventually, the Iwate Co-op held the salon independently.

### 4.3 Effects of the activities

The effects of the cooking classes conducted by Iwate Co-op in cooperation with TAF are classified into the following three points. First, owing to the encounter and collaboration with TAF, the cooking class was continuously conducted even after TAF withdrew from the disaster-affected areas. The cooking class project was a form of activity development initially undertaken by the Iwate Co-op. They learned from each other while cooking, enjoyed the food together, and talked to each other after class. Owing to this background, they were able to build a new community network even when they moved from temporary housing to disaster-affected public housing (24).

Second, the Iwate Co-op recruited and trained new staff members to run the cooking classes independently. In addition, the Iwate Co-op was actively involved not only in obtaining the necessary budget for the operation, but also in planning, implementing, and evaluating the activities, which led to the activation of member activities in an independent manner.

Third, they developed the cooking class project in inland areas other than the disaster-stricken areas based on the assumption that they could infuse the project into their existing activities, such as creating a place for older people, as food problems affect people beyond disaster victims.

A total of 88 cooking classes were held in cooperation with TAF over 3 years and 5 months, with 651 participants. Of these, 60 were held by the food support staff on their own initiative, with 429 participants.

## 5 Discussion

As an outcome evaluation of the food support project that Iwate Co-op and TAF collaborated on, it was found that in the short term, connections with fellow members increased, food awareness and health behaviors improved, and the utilization component of food security improved (24). In the long term, community resilience was strengthened as a result of the collaboration with TAF, which resulted in the activation of voluntary membership activities, expansion of active areas, revitalization of the community, and increased solidarity. However, the sustainability of community resilience requires continued follow-up.

The Iwate Co-op elaborately organized cooking classes to meet community needs under community-led initiatives. A good partnership with TAF ensured the program’s sustainability. The classes helped foster social bonds and a support network during the transition from temporary to permanent housing. Residents’ active participation and the recruitment of “food lovers” strongly emphasized shared responsibility. The Co-op provided diverse dietary needs, ensuring all community members could benefit. They transitioned from relying on TAF to developing their own staff and curriculum, promoting the importance of continuous learning and adaptation. The Iwate Co-op has played an essential role in the local community by utilizing experiences and knowledge gained from the project to develop sustainable community-based support activities.

The collaborative food support project by the Iwate Co-op and TAF contributed to the resilience of the affected people and communities by strengthening bonds and solidarity among residents and organizations. The Iwate Co-op could develop self-reliant activities to “create their own future,” while TAF worked on the philosophy “To contribute to the development of countries and regions all over the world and the creation of a bright future for the people living there through our food and nutrition activities.” The key to success was the fusion of a traditional sense of independence in the co-op (25) with TAF’s mission (26) and know-how.

This case study included several elements that have been identified in previous research as effective for community and individual recovery from disasters. These were community-led efforts (27, 28), multi-organizational collaboration (29, 30), mutual aid and co-help (31, 32), recognition of community responsibilities and roles and shared interrelationships (33, 34), social cohesion and cooperation (7), and an emphasis on social justice and equity (35).

This case study has several limitations. This case is an analysis of the collaboration between Iwate Co-op and Ajinomoto Foundation only. Further analysis of other local cases would provide a better understanding of community resilience. In addition, long-term validity and sustainability for community resilience requires deliberate consideration and continual improvement. These findings provide an excellent example of post-disaster community resilience, which can be applied to future disasters.

## 6 Conclusion

The cooking class activities with collaborative efforts by TAF and the Iwate Co-op contributed to the resilience of the community in the Iwate Prefecture following the Great East Japan Earthquake. The three effects of collaboration were continued support for disaster victims through food support projects, independent activation of member activities, and the revitalization of the region. The project activities not only strengthened the bonds and solidarity among residents and organizations, but also accelerated the resilience of the affected people and communities. The Iwate Co-op plays an essential role in the local community by developing sustainable community-based support activities. This report presents an example of post-disaster community resilience and provides a reference for future disasters.

## Data availability statement

The original contributions presented in the study are included in the article/supplementary material, further inquiries can be directed to the corresponding author.

## Ethics statement

Written informed consent was obtained from the individual(s) for the publication of any potentially identifiable images or data included in this article.

## Author contributions

NI: Methodology, Conceptualization, Formal analysis, Writing – original draft, Writing – review & editing. KKo: Investigation, Writing – review & editing. KN: Investigation, Writing – review & editing. KF: Investigation, Writing – review & editing. KKa: Investigation, Writing – review & editing. HK: Conceptualization, Writing – review & editing. YN: Conceptualization, Writing – review & editing. MY: Conceptualization, Visualization, Writing – review & editing. AK: Methodology, Conceptualization, Data curation, Investigation, Formal analysis, Project administration, Writing – original draft, Writing – review & editing. TK: Methodology, Conceptualization, Formal analysis, Writing – review & editing. YK: Methodology, Conceptualization, Data curation, Investigation, Formal analysis, Project administration, Writing - original draft, Writing – review & editing, Funding acquisition. TN: Supervision, Writing – review & editing. YF: Project administration, Supervision, Writing – review & editing.
